# Supplementary material for: Total Soil CO2 Efflux from Drained Terric Histosols
Source: Plants (Basel). 2024 Jan 4;13(1):139. doi: 10.3390/plants13010139 (PMC10780588; doi:10.3390/plants13010139)
Supplement: Supplementary file 1 [file plants-13-00139-s001.zip › plants-2801491-supplementary.pdf]

Table S1. Mean dendrometric properties of the studied forest stands.

Tree species	Age, yr	H, m	DBH, cm	Tree density, Trees ha^{-1}	Stem volume, m^3 ha^{-1}
Norway spruce	70	19.3	20.2	860	259
Silver birch	43	18.0	20.4	893	193
Black alder	30	18.8	16.5	1600	238

Notes: DBH – diameter at breast height (1.3 m); H – height of tree.
